# Ultrathin Gallium Nitride Quantum-Disk-in-Nanowire-Enabled Reconfigurable Bioinspired Sensor for High-Accuracy Human Action Recognition

**DOI:** 10.1007/s40820-025-01888-w

**Published:** 2025-09-01

**Authors:** Zhixiang Gao, Xin Ju, Huabin Yu, Wei Chen, Xin Liu, Yuanmin Luo, Yang Kang, Dongyang Luo, JiKai Yao, Wengang Gu, Muhammad Hunain Memon, Yong Yan, Haiding Sun

**Affiliations:** 1 iGaN Laboratory, School of Microelectronics, University of Science and Technology of China, Hefei, 230029 People’s Republic of China; 2 Institute of Materials Research and Engineering, 2 Fusionopolis Way, #08-03Agency for Science Technology and Research, Singapore, 138634 Singapore

**Keywords:** GaN nanowire, Quantum-confined Stark effect, Voltage-tunable photoresponse, Bioinspired sensor, Artificial vision system

## Abstract

Human action recognition (HAR) is crucial for the development of efficient computer vision, where bioinspired neuromorphic perception visual systems have emerged as a vital solution to address transmission bottlenecks across sensor-processor interfaces. However, the absence of interactions among versatile biomimicking functionalities within a single device, which was developed for specific vision tasks, restricts the computational capacity, practicality, and scalability of in-sensor vision computing. Here, we propose a bioinspired vision sensor composed of a GaN/AlN-based ultrathin quantum-disks-in-nanowires (QD-NWs) array to mimic not only Parvo cells for high-contrast vision and Magno cells for dynamic vision in the human retina but also the synergistic activity between the two cells for in-sensor vision computing. By simply tuning the applied bias voltage on each QD-NW-array-based pixel, we achieve two biosimilar photoresponse characteristics with slow and fast reactions to light stimuli that enhance the in-sensor image quality and HAR efficiency, respectively. Strikingly, the interplay and synergistic interaction of the two photoresponse modes within a single device markedly increased the HAR recognition accuracy from 51.4% to 81.4% owing to the integrated artificial vision system. The demonstration of an intelligent vision sensor offers a promising device platform for the development of highly efficient HAR systems and future smart optoelectronics.

**Supplementary Information:**

The online version contains supplementary material available at 10.1007/s40820-025-01888-w.

## Highlights

- A novel GaN/AlN-based ultrathin quantum-disks-in-nanowires sensor was fabricated, demonstrating voltage bias tunable response characteristics to light stimuli.
- Image enhancement functionality and a robust reservoir computing system were demonstrated based on the voltage tunable long-term and short-term persistent photocurrent respectively.
- Furthermore, a high-performance artificial vision system with the two integrated functions was demonstrated, achieving a remarkable improvement in human action recognition.

**Supplementary Information:**

The online version contains supplementary material available at 10.1007/s40820-025-01888-w.

## Graphical abstract

**Figure:**
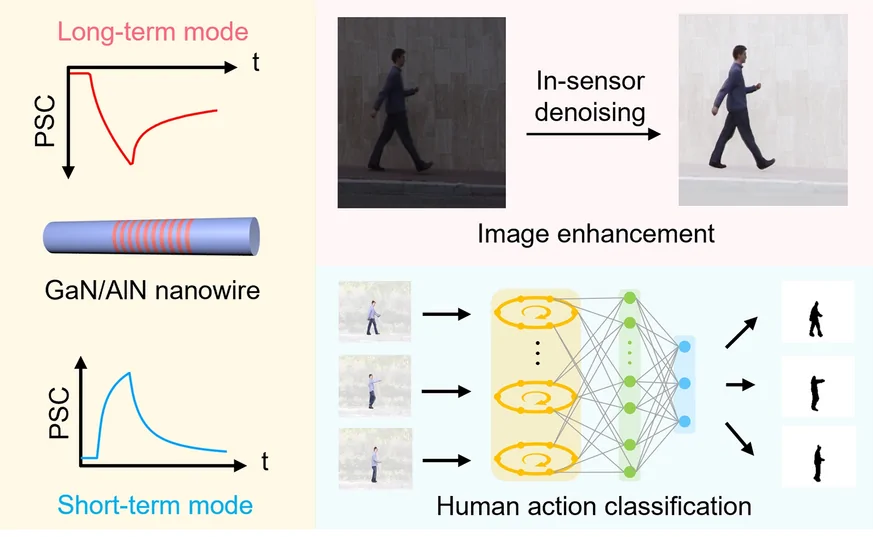

## Introduction

In the era of technological revolution, human action recognition (HAR) technology, characterized by artificial intelligence, has become increasingly important in various applications, including security surveillance, video retrieval, human–computer interaction, and autonomous navigation [1–7]. However, HAR from video sequences still faces challenges, such as background clutter, partial occlusion, variations in scale or viewpoint, lighting conditions, and appearance changes [8–10]. To date, long short-term memory (LSTM) architectures have been successfully applied to analyze temporal complex human activity data because of the recurrent connections in their hidden layers [11–13]. However, these approaches also have several drawbacks, such as the need for large datasets and the time- and energy-consuming training process [14].

In contrast, biological vision systems can efficiently and autonomously perceive motion-related information, performing image enhancement and classification tasks in real time through the coordinated operation of various retinal cells, including photoreceptors, bipolar cells, and ganglion cells [15–18]. Notably, retinal ganglion cells can be categorized into two types: magnocellular (Magno) and parvocellular (Parvo). Magnocellular cells respond rapidly in motion detection scenarios, corresponding to “short-term” functionality, whereas parvocellular cells exhibit a slower response in low-contrast environments, corresponding to “long-term” functionality [19–21]. Consequently, the human visual system exhibits remarkable adaptability to a wide range of environmental conditions—whether static or dynamic, and from blurred to well-defined stimuli—enabled by biologically inspired long short-term memory (bio-LSTM) architectures. This functional differentiation among retinal cells underpins the efficiency of natural vision and serves as inspiration for the development of dual-functional artificial vision devices.

Herein, we propose a versatile vision sensor composed of GaN/AlN-based ultrathin quantum-disks-in-nanowires (QD-NWs) with reconfigurable photoelectric properties to mimic visual behaviors in biological cells. Notably, the well-designed nanowire consists of an n-GaN layer, GaN/AlN multiple QDs, and an n-GaN cap layer. The *n*–*i*–*n*-type band structure minimizes the separation between electrons and holes, whereas the high barrier height of the AlN quantum barriers confines carriers in the QD-NWs, enabling a dual-modal persistent photocurrent (PPC). Furthermore, each quantum disk comprises several layers of GaN, which enhances the quantum-confined Stark effect (QCSE) and spontaneous polarization. This design allows for modulation of the wavefunction overlap, which regulates the recombination probability of nonequilibrium carriers, enabling the PPC behavior to switch between the “long-term mode” and “short-term mode”. As a result, the device can carry out image sensing and preprocessing tasks very well apart from fundamental synaptic plasticity performance under long-term mode. Additionally, a high-performing and robust long short-term reservoir computing (LSTRC) system was constructed based on QD-NWs for human action recognition in short-term mode. Finally, an integrated artificial vision system is constructed with a remarkable improvement in recognition accuracy from 51.4% to 81.4%. The advancement of the proposed QD-NW bioinspired vision sensor holds significant promise for the development of compact and efficient artificial vision systems.

## Experimental Section

### Epitaxy

The nanowires used in this work were grown on planar *n*-type Si substrates via plasma-assisted molecular beam epitaxy. Before the Si wafers were loaded into the molecular beam epitaxy chamber, they were cleaned with acetone, methanol, and HF-H_2_O solution to remove organic contaminants and surface oxides. Thereafter, to further remove the organic contaminants and water components, the Si wafers were outgassed in the buffer chamber at 780 °C before growth initiation. Then, during nanowire growth, nitrogen radicals were supplied from a radio-frequency plasma source. The Al, Ga, Mg, and Si fluxes were controlled by the respective thermal effusion cells. The detailed growth process followed previous works [22–24].

### Device Fabrication

The samples were thoroughly cleaned with acetone, isopropyl alcohol (IPA), and deionized (DI) water. Subsequently, 20% HF was used to remove surface oxidation from the NWs. Thin metal stacks of Ti/Au (5/5 nm) were then deposited on the top of the NW array. The thin film showed a 33.1% transmittance at the wavelength of 254 nm which suggests its semitransparent feature (Fig. S1). During the metal evaporation process, the NW wafers were tilted ≈ 40° (Fig. S2). Finally, Ti/Au (10/150 nm) stacks were deposited on the back side of the samples (Si side) to achieve back contacts.

### Characterizations

The nanowires for STEM characterization were mechanically removed from the epitaxial Si substrates and dispersed on a lacy carbon film mesh Cu TEM grid. The STEM measurements were conducted using an FEI Talos F200X instrument operating at 200 kV. The nanowires have an average length of ~ 300 nm with *μ* = 307.5 nm, *σ* = 11 nm, as characterized by 100 nanowires and the statistical data are shown in Fig. S3. The *I*–*V* characteristics were measured via an Agilent B1500A semiconductor device analyzer.

### FDTD Simulation

In FDTD modeling for simulating the behavior of light within a device, the refractive indices and absorption coefficients for GaN can be obtained from previous works [25, 26]. The 254 nm plane wave sources are placed at the top of the NWs.

### Human Action Classifications

The Weizmann Human Action Dataset was used for the human action classification task, which included 10 human actions recorded from the performance of 11 people under different lighting conditions; these actions included running, walking, skipping, jumping jack (jack), jumping forward on two legs (jump), jumping in place on two legs (pjump), gallop sideways (side), wave two hands (wave2), wave one hand (wave1), or bending. We used the foreground-mask videos in the dataset, clipped the videos into a four-frame clip by sliding a clipping window of four frames through each video, and average-pooled them into 15 × 12-pixel data for RC input.

## Results and Discussion

### Two Types of Ganglion Cells and Reconfigurable Artificial Visual Sensor

**Fig. 1:**
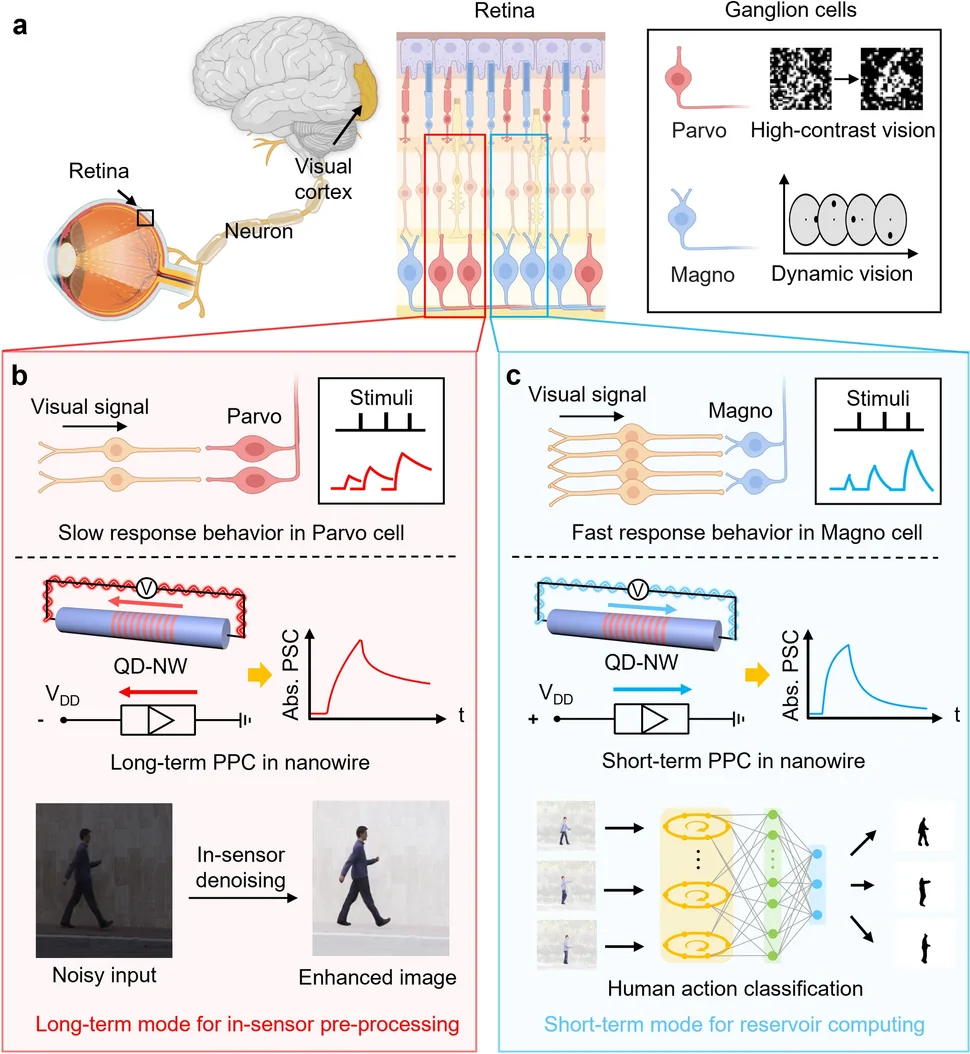
Schematic of the human vision system and nanowire-based neuromorphic device with long-term mode for in-sensor image enhancement and short-term mode for high-level data classification. **a** In the human visual system, visual information is sensed by photoreceptors and then preprocessed by ganglion cells, and the processed images are transmitted to the visual cortex for high-level processing, including recognition and classification. Owing to their ability to undergo cell differentiation, ganglion cells present profoundly different response behaviors, including fast response and slow response characteristics, to the input signals from photoreceptors. Similarly, our proposed intelligent humanoid vision sensor can operate in two modes: **b** Long-term mode under negative bias for image sensing and preprocessing and **c** short-term mode under positive bias for reservoir computing

The human visual system, characterized by a hierarchical biostructure, comprises the retina, optic nerve, and visual cortices [27]. The visual information is initially sensed by the photoreceptor cells and subsequently processed with the assistance of the nerve layer, including bipolar cells, horizontal cells, and ganglion cells, facilitating a highly efficient vision system, as illustrated in Fig. 1a. Notably, ganglion cells can be divided into magnocellular and parvocellular cells via cell differentiation and are distinguishable both anatomically and physiologically [20]. The Magno cells are larger and exhibit faster responses, indicating their role in motion detection. In contrast, parvo cells are smaller and respond more slowly to input signals, playing a key role in low-contrast vision [21]. These distinct visual characteristics enhance image processing and motion classification while utilizing limited computational resources.

Inspired by the dual-functional nature of ganglion cells, we developed a GaN nanowire-based vision sensor. Owing to its efficient bias-tunable PPC characteristics, the NW sensor exhibits long-term PPC under “Parvo mode” for sensing and in-sensor image preprocessing (Fig. 1b; details are shown in Sect. 3.3). In addition, a high-performing LSTRC system for human action classification based on the NW sensor was also demonstrated under “Magno mode”, which revealed short-term PPC behavior (Fig. 1c; details are shown in Sect. 3.4). These two adjustable response features to light stimuli closely align with the characteristics of the biological vision system, and a high-performing functional fusion artificial vision system was constructed for human action classification.

### Characterization of the Nanowire Structure and Light-Triggered Photoelectric Performance of the Device

**Fig. 2:**
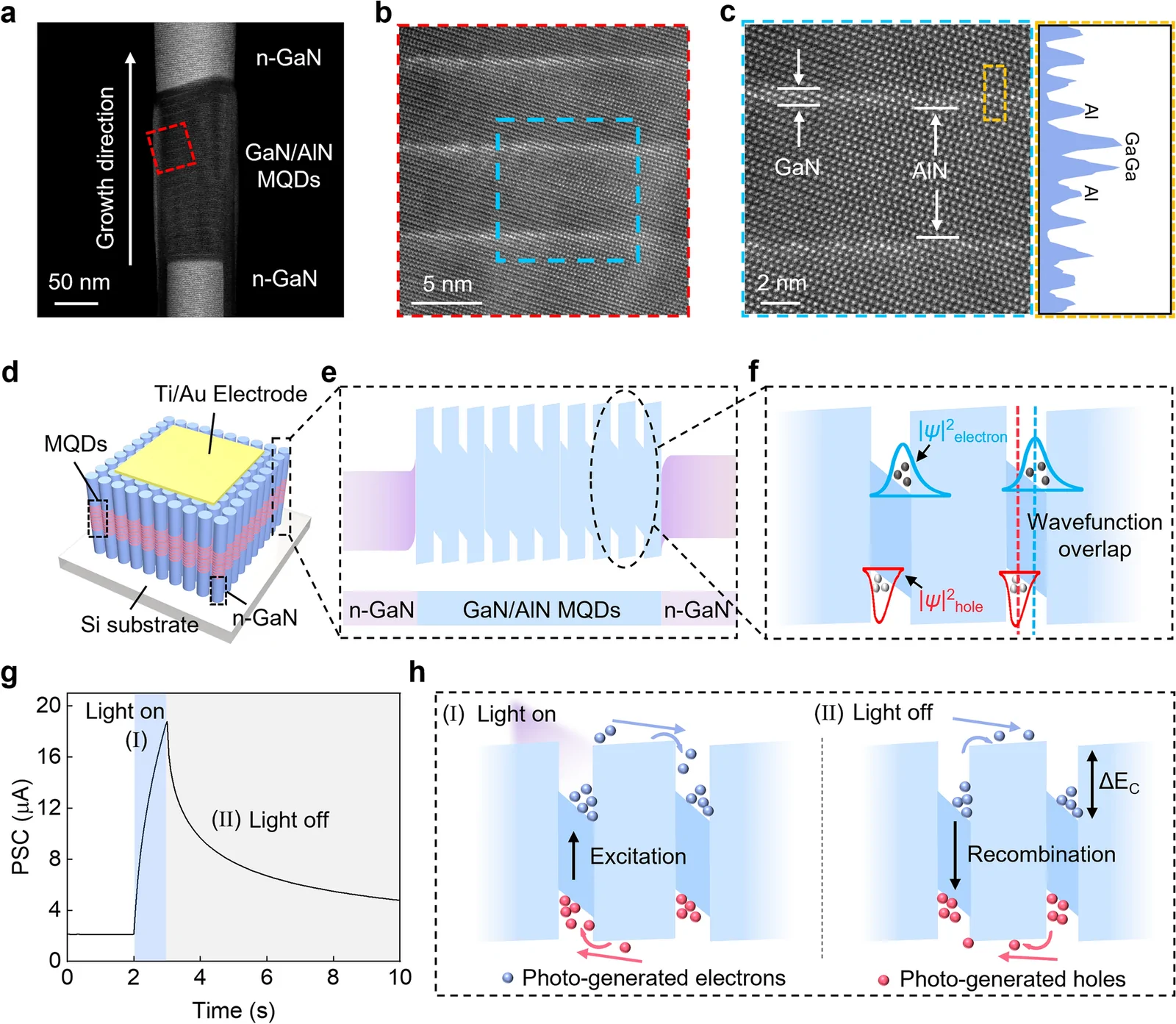
Schematic and characterization of the nanowire and device. **a** HAADF-STEM image of a single NW with an *n*-type GaN layer, GaN/AlN MQDs, and an *n*-type GaN cap layer. **b** Partial enlargement image of the slope section near the edge of the NW. **c** Atomic image of the GaN quantum disks separated by AlN quantum barriers and an atomic model corresponding to the schematic of the crystal lattice. **d** Vertically structured optoelectronic device schematic based on the designed nanowires. The electrode size was defined as 200 × 200 μm^2^. **e** Band structure diagram of the nanowire. **f** Detailed view of the quantum structures in the active region. **g** Persistent photocurrent observed in the NW sensor under 254 nm light illumination. **h** (I) Structure and band alignment of the nanowire under positive bias and 254 nm illumination, illustrating the photocurrent generation mechanism. (II) Carrier transfer mechanism after UV light illumination

The designed nanowire sensor serves as the fundamental building block of the bioinspired vision system. Therefore, detailed characterization of its microstructure is essential for understanding and optimizing device performance. The GaN-based NWs were directly grown on the n-Si substrate and feature a multilayer structure, including an *n*-type GaN layer, GaN/AlN MQDs, and an *n*-type GaN cap layer. GaN-based nanowires are selected for their exceptional optoelectronic properties, including the tunable bandgaps of the GaN material system, as well as the advantages provided by their one-dimensional (1D) geometry, such as strain relaxation, a large surface-to-volume ratio, and CMOS compatibility when grown on silicon substrates [28–30]. As illustrated in Fig. S4, energy-dispersive spectroscopy (EDS) element mapping demonstrated the distribution of Ga, Al, and N atoms, further revealing that the nanowires were grown under precise control. To confirm the successful growth of the GaN/AlN heterostructures, high-angle annular dark-field scanning transmission electron microscopy (HAADF-STEM) was performed, as shown in Fig. 2a. The dark-field image of a single nanowire was divided into three parts, reflecting the different contrasts of gallium and aluminum atoms. Notably, the MQDs presented slopes close to the nanowire sidewalls, corresponding to the diffusion-controlled growth mechanism of III-nitride nanowires and the differences in incorporation efficiency on different crystalline planes [31, 32]. In Fig. 2b, the darker contrast indicates the AlN barriers, whereas the brighter lattice contrast represents the GaN disks, and the statistics of the GaN layer thicknesses are in good agreement with the designed values. The GaN layers can be recognized in the high-magnification atomic-resolution image (Fig. 2c). To determine the composition of the GaN layer, the line intensity profiles were further revealed, revealing several layers of GaN embedded within the AlN. The energy band structure was analyzed via Advanced Physical Models of Semiconductor Devices (APSYS) provided by Crosslight, Inc. Figure 2e shows the simulated band structure of the nanowire, clearly delineating its three constituent regions. A magnified view of the quantum disk region is provided in Fig. 2f. Owing to the differences in spontaneous and piezoelectric polarization between GaN and AlN, the band structure exhibits a characteristic sawtooth-like profile. Notably, obvious piezoelectric and spontaneous polarizations in quantum wells under the equilibrium state and electron and hole wavefunctions (|*ψ*|^2^) are localized in the GaN disk region [33, 34]. Polarization-field-induced separation of electrons and holes reduces the degree of overlap of the wavefunctions, thus reducing the probability of electron and hole recombination. The *n*–*i*–*n* structure, with *n*-type GaN on both sides of the intrinsic (GaN/AlN MQD) region, ensures symmetry in carrier injection and extraction. Additionally, the GaN/AlN heterostructure configuration supports better quantum confinement and separation of photogenerated carriers within the quantum disks, while maintaining effective confinement within the MQDs for PPC generation, which is critical for realizing the bias-tunable persistent photocurrent behavior. The well-designed architecture thus enables promising applications in advanced photoelectronic systems.

To investigate the photoelectric performance of the nanowire, a vertical structure device was fabricated, with the fabrication process detailed in the methods section and illustrated in Fig. S2. A 5/5 nm Ti/Au layer was deposited atop the NW array as the top contact pad. For the back side, a 10/150 nm Ti/Au layer served as the back contact pad. A schematic of the device is shown in Fig. 2d, and the current–voltage sweeping curve under various light intensity is shown in Fig. S5, demonstrating the photoresponsivity of the QD-NW device. As shown in Fig. 2g, when a positive bias and 254 nm deep ultraviolet (DUV) illumination are applied to the device, a photocurrent is generated and gradually increases, corresponding to process (I). Notably, upon removal of the light source, the photocurrent does not immediately vanish but instead decays slowly over time, leading to the emergence of a persistent photocurrent (PPC), as illustrated in process (II). Figure 2h (I) shows the band alignment of the nanowire under positive bias and UV light stimuli. According to the results of 3D finite-difference time-domain (3D-FDTD) calculations (Fig. S6), DUV light at 254 nm is predominantly absorbed by the MQD region of the NWs. Under 254 nm illumination, electrons and holes are excited and subsequently separated by the external electric field, leading to the generation of a DUV light-induced photocurrent. After illumination (Fig. 2h(II)), the photoinduced electrons and holes become trapped in the GaN disks due to the high energy barrier of AlN, causing the slow decay of the photocurrent, corresponding to the PPC phenomenon. Moreover, the slow photonic response exhibited by the device emulates the biomimetic capture and release of neurotransmitters, reflecting the changes in synaptic weight observed in bionic synapses. The nanowire device exhibits bioinspired functionalities similar to those of biological visual systems, particularly in terms of differential light adaptation and memory behavior. Accordingly, a mechanistic analysis of the voltage-modulated photoresponse characteristics is provided to elucidate the underlying physical principles.

### Image Acquisition with Enhanced Performance in Long-Term Mode

**Fig. 3:**
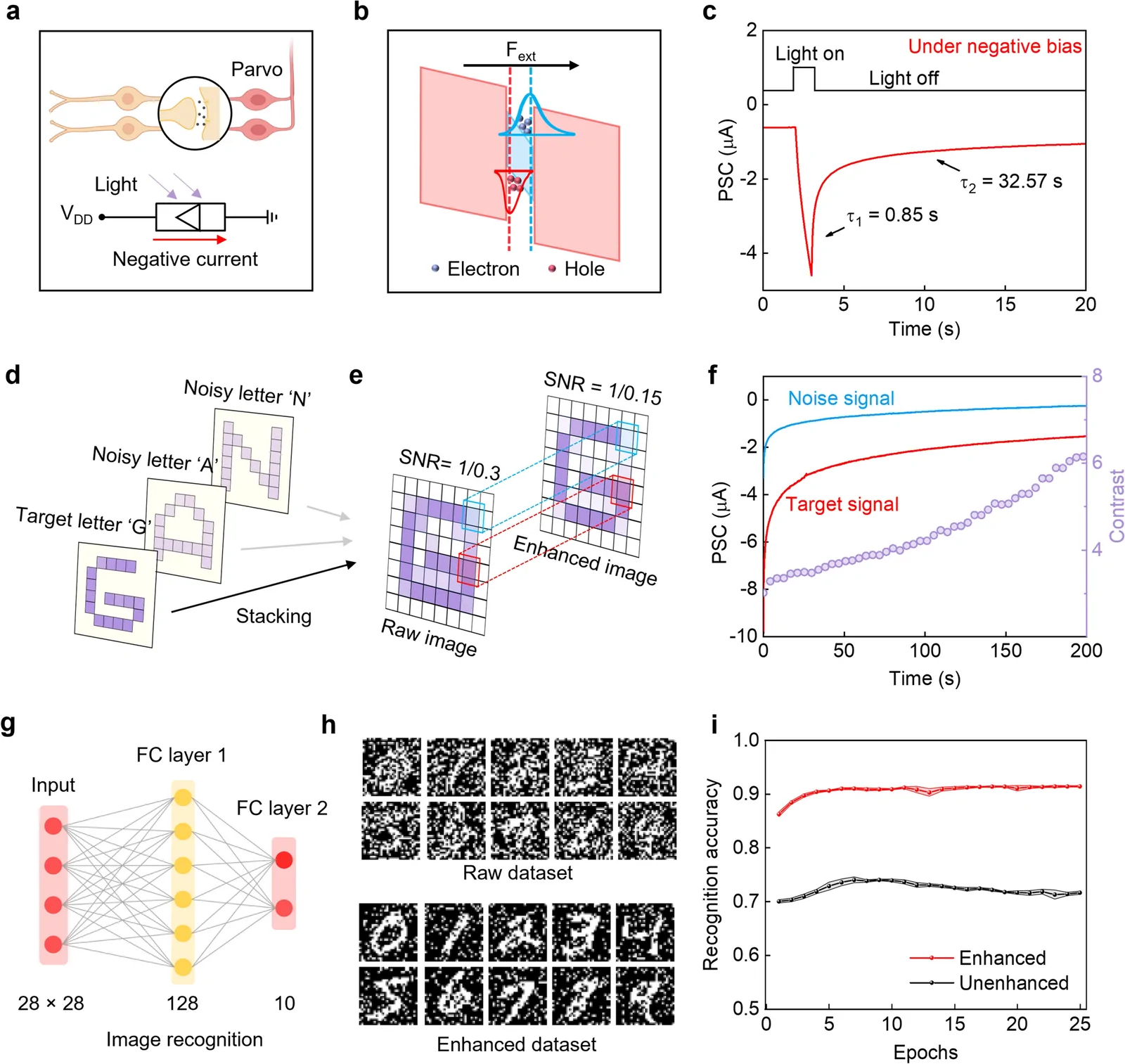
Long-term PPC behavior-based image enhancement. **a** Schematic diagram of the proposed sensor under negative bias and a slow response to light illumination, corresponding to the biological behavior of Parvo cells. **b** Polarization degree enhancement under negative bias, reducing the wavefunction overlap. **c** Biomimetic long-term persistent photocurrent phenomenon observed in the nanowire device under 254 nm UV light illumination and negative voltage bias. **d** Schematic of the main letter and noisy letter stacking and a vague image as the input to the NW sensor. **e** In-sensor preprocessed image with the enhanced main letter “G.” **f** Current decay characteristics triggered by different light intensities and increasing contrast over time. **g** Schematic diagram of the constructed artificial neural network for image recognition (FC layer 1: ReLU activation; FC layer 2: Softmax activation). **h** Comparison of images before (SNR = 1/0.3) and after enhancement (SNR = 1/0.15). **i** Recognition accuracy over training epochs of the NW sensor array for image preprocessing (shaded area: std. *N* = 5)

When the NW device is negatively biased (Fig. 3a), the external electrical field direction is aligned with that of the polarization field, and the GaN disk energy band tends to be more inclined. Thus, the inclination enhances more separation of electrons and holes in the wells, reducing the degree of wavefunctions overlap (Fig. 3b). Consequently, the probability of recombination for nonequilibrium carriers generated by UV light decreases, leading to a relatively long PPC (Fig. 3c). The decay process can be well fitted by an exponential function with two relaxation times:

1 $$I = A_{1} e^{{ - \frac{\Delta t}{{\tau_{1} }}}} + A_{2} e^{{ - \frac{\Delta t}{{\tau_{2} }}}} + I_{0}$$

where *I* represents the photocurrent, *A*_1_ and *A*_2_ are the fitting prefactors, and *τ*_*1*_ and *τ*_2_ denote the time constants associated with the rapid and slow relaxation phases, respectively. *I*_0_ is the steady-state value of the photocurrent. *τ*_1_ and *τ*_2_ are extracted from the formula to be 0.85 s and 32.57 s, respectively, which demonstrates an initial fast decay followed by a slow decay. Notably, the kinetics of current decay closely resemble the memory loss behavior observed in neuronal systems [35]. Band structures under different bias voltages are simulated, as shown in Fig. S7. When the bias is switched from − 3 V to 0 V, the degree of wavefunction overlap changes from 69.5% to 76.2%. The experimental data in Fig. S8 also corroborate the simulation results. The PPC behavior varies as the bias voltage changes from − 2 V to − 0.5 V. The PSC decay processes are fitted by formula (1), and the fitting parameter τ_2_, which represents the long-term decay phase, has relatively long duration characteristics and decreases from 35.94 s to 28.05 s as the degree of polarization reduction caused by the external bias voltage. Additionally, the repeatability and uniformity of the PPC behaviors are essential to high-performance and reliable voltage bias-tunable functions. As a result, the repetitive experimental data from stochastically selected 20 QD-NW devices were characterized, as shown in Fig. S9a, illustrating the good voltage bias adjustable decay time reproducibility. Also, we switched on and off the light 20 times under the same bias voltage conditions, the representative device shown good cycle-to-cycle variation, as shown in Fig. S10 red point plot.

In this scenario, pulse interval-dependent plasticity was demonstrated by applying two successive light pulses with a duration of 1 s and different interval durations under a negative bias, analogous to the paired-pulse facilitation (PPF) observed in biological synapses, as shown in Fig. S11a inset. In biological systems, PPF is a crucial short-term plasticity enhancement process and is essential for the temporal decoding of visual signals. PPF can be defined as (*A*_2_/*A*_1_) × 100%. The PPF index strongly depends on the pulse interval (Δ*t*), where the distribution of the PPF index can be well fitted by a double-exponential equation [36, 37]:

2 $${\mathrm{PPF}}\;{\mathrm{index}} = C_{1} e^{{ - \frac{{\Delta t}}{{\tau _{1} }}}} + C_{2} e^{{ - \frac{{\Delta t}}{{\tau _{2} }}}}$$

The fitting curve indicates the exponential fitting result, as shown in Fig. S11a. The PPF index decreases from 184% to 125% as Δt increases from 0.5 s to 15 s after applying paired-light pulses. This is consistent with more recombination of the confined carriers in the GaN disks with longer Δ*t*. The facilitation process can be categorized into fast and slow decay components, depending on the comparison between Δ*t* and the PSC decay time. When Δ*t* is much less than the decay time, the PPF index decreases rapidly with Δ*t*, corresponding to the fast decay process featuring a small *τ*_1_. As Δ*t* is sufficiently large, the PSC increment evoked by the second spike is limited, implying that more carriers relax during longer intervals, resulting in the PPF index gradually approaching 100% as Δ*t* increases, which is consistent with previously reported neuro-inspired optically stimulated devices [38–41]. Furthermore, on the basis of the PPF behavior, the synaptic plasticity transition characteristics are also achieved in such a long-term mode situation, as shown in Fig. S12.

Vision serves as the primary channel through which humans acquire external information, making visual memory the most efficient mode of memory within the human brain [42]. Consequently, artificial visual sensors designed for constructing intelligent vision systems should demonstrate robust visual memory behaviors akin to those observed in the brain. A neural-inspired optical sensor array was constructed on the basis of 8 × 8 matrix nanowire pixels (Fig. S13). The device-to-device variation was statistically evaluated (Fig. S14). The excellent uniformity of performance metrics of the device is crucial for the acquisition of high-quality images in the sensing system.

The effective modulation of the current relaxation speed was also determined in the nanowire sensor by adjusting the intensity of light stimulation, thus enabling the experimental implementation of image preprocessing, particularly in enhancing contrast between the target and background letters, a fundamental function in bio-vision compared with a digital imaging system. To evaluate the performance of the neuro-inspired optical sensor array, a “G”-shaped photomask with 17 pixels was placed on top of the array for measurement (measurement setup is shown in Fig. S15). The light transmitted through the photomask and then focused by the lens. The pattern on the mask was projected onto the device and the photocurrent of each pixel was recorded. Initially, we projected the target letter onto the sensor array via an optical mask at a light intensity of 0.985 mW cm^−2^ and recorded the corresponding current of each pixel. The photocurrent of the noisy letter with a light intensity of 0.553 mW cm^−2^ was also obtained via the same procedures. Figure 3d shows the light intensity map of the input image corresponding to the photocurrent after 1 s of illumination, including a target letter “G” and relatively faint noisy letters “A” and “N.” After decay for 200 s, the remaining photocurrents of the pixels were extracted and map-plotted (Fig. 3e). Figure 3f displays the photocurrent decay characteristics of the device triggered by the same light pulses with light intensities of 0.985 and 0.553 mW cm^−2^. Since the current triggered by a lower light intensity decays faster, the current triggered by a higher light intensity decays more slowly. The corresponding average values of the pixels in the target letter and noisy letter are normalized as the signal-to-noise ratio (SNR). Compared with the light intensity contrast of the input image, the current contrast between pixels with different light illuminations is increased from SNR = 1/0.33 to 1/0.15 after the decay process without the use of external circuits, just like when people focus on a specific object, the remaining surrounding information is mostly filtered. Thus, a considerable increase in the ratio of the remaining photocurrent contrast is estimated to be 6.6 after decay process. The light intensity-dependent current relaxation speed enables contrast enhancement of the input image, aligning with the transition from STP to LTP corresponding to the light pulse intensity, enabling highlighting of the target letter within a noisy background. In contrast to traditional image sensors, which rely on light signal inputs translated into electrical outputs before software postprocessing, our bioinspired nanowire devices offer a more integrated approach. They can directly generate synaptic outputs upon receiving optical signals, thereby executing image enhancement tasks in real time without the need for additional filters or postprocessing software. Clearly, the enhanced image after repeated training demonstrates an enlarged difference between the grayscale of the pixels over the input images, thus contributing to an output image with enhanced contrast and highlighted features. To evaluate the recognition accuracy of the images before and after contrast enhancement, we constructed a simple artificial neural network with only one hidden layer without any preprocessing function, as shown in Fig. 3g, benchmarked by a 70,000-sized image dataset (60,000 for training and 10,000 for testing), including 10 categories of noisy handwritten letter images from the Modified National Institute of Standards and Technology (MNIST) dataset. Each image with a noise rate = 0.3 was extracted as the raw dataset, and images with a noise rate = 0.15 were extracted as the enhanced dataset for image recognition. A comparison of the raw image dataset and the enhanced dataset indicated that preprocessing of the sensor array evidently reduced the noise, as depicted in Fig. 3h. The recognition accuracy of the images is shown in Fig. 3i. A remarkable improvement in the recognition accuracy was achieved from 71.6% to 91.4% after enhancement with only 25 training epochs. The results of the enhanced output image indicated that the NW sensor array could have high competitiveness in intelligent image sensing systems under negative bias.

### Reservoir System based on the QD-NW Sensor for Human Action Classification in Short-Term Mode

**Fig. 4:**
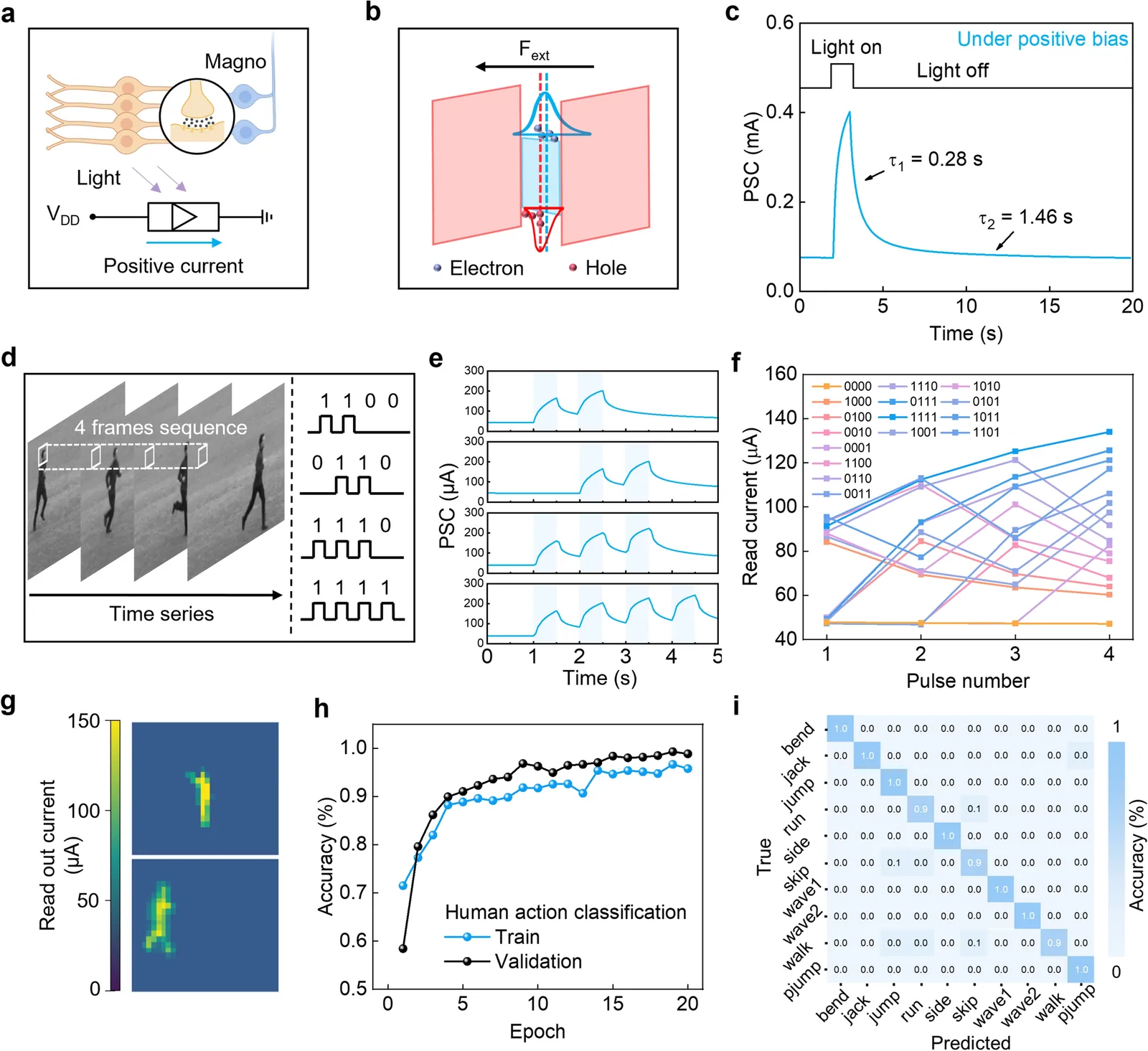
Short-term PPC behavior-based reservoir computing for human action classification. **a** Schematic diagram of the proposed sensor under positive bias and a fast response to light illumination, corresponding to the biological behavior of Magno cells. **b** Polarization attenuated under positive bias, increasing the wavefunction overlap. **c** PPC phenomenon under 254 nm UV light illumination and positive bias. **d** Input temporal signals of the video classification task and four frames extracted from the video and coded into four bits of light pulses. **e** Photoresponse characteristics and input–output feature extraction of four representative inputs: “1100,” “0110,” “1110” and “1111.” **f** Readout current generated by 16 series of optical pulse trains ranging from “0000” to “1111.” **g** Reservoir output for a sample of “wave1” and “run” from the Weizmann dataset. The results show that the reservoir array successfully retains four frames of the action stream. **h** Training and validation recognition accuracy of the as-built in-sensor RC system. **i** Confusion matrix for classifying the 10 human actions. (The number of significant decimal places for the accuracy value was set to 1)

With the demonstration of the short-term mode, the QD-NW sensor has been able to achieve contrast vision well. More interestingly, as mentioned previously, the Magno cells respond quickly to neural signal stimuli, and similarly, the QD-NW sensor shows a fast response to light stimuli (Fig. 4a). When a positive bias is applied to the device (Fig. 4b), the internal polarization direction is opposite to the external electric field, resulting in a reduction of the overall polarization field. Under these conditions, the overlap of electron and hole wavefunctions increases, enhancing the recombination rate of nonequilibrium carriers and giving rise to a short-term PPC effect. Although the photocurrent exhibits a similar response under negative bias, the PPC duration in the positively biased case is noticeably shorter. The simulation results also show that the degree of wavefunction overlap differs with the applied voltage, ranging from 76.2% to 79% as the bias changes from 0 V to 3 V, as shown in Fig. S7. The experimental data in Fig. S8 are also consistent with the simulation results. The PPC behavior varies as the bias voltage changes from 0.5 V to 2 V. The fitting parameter *τ*_2_ decreases from 4.95 s to 1.46 s with decrease in polarization degree caused by the external bias voltage and presents short-term behavior overall compared with the situation under negative bias. The repeatability and uniformity experiments were also constructed (Figs. S9b and S10 blue point plot), showing good controllability of our QD-NW device. Similarly, the fundamental PPF and STP-to-LTP transition behaviors are successfully achieved by adjusting the illumination conditions, as shown in Figs. S11b and S16.

In short-term mode, a hardware-emulated reservoir computing system based on the QD-NW device is constructed. On the basis of the four-bit reservoir capacity of our device, it can be modeled on biological systems to implement in-sensor RC for human motion classification. In the case of running motion, for example, four optical frames (15 × 12 scale) in continuous motion are considered as input data and then enter the reservoir array in time series without any analog-to-digital conversion. To clearly describe the input process, representative pixels of the four frames are highlighted with white boxes. The pixels at the same position in the four frames are converted into four light pulses according to the binary colors and then fed into a reservoir in a time sequence (Fig. 4d). To illustrate the feature sampling, the *I*–*t* curves of four representative inputs of “1100,” “0110,” “1110,” and “1111” of the QD-NW reservoir are shown in Fig. 4e. Although the valid last pulses are all “1,” their decay processes after the input sequences are different. Therefore, the final state of the reservoir not only is related to the last input but also depends on its real-time state, indicating the lateral connections in such a nanowire reservoir. The 10 human actions from the Weizmann Human Action Dataset for the spatiotemporal recognition task were applied for performance evaluation [43]. Details about the dataset are provided in the Methods section. The foregrounded mask video set was used in this task. Here, the reservoir arrays sense the light sequences and transmit them to the reservoir states connected to the input neurons for classification. To demonstrate the capability of the feature mapping of the reservoir, a four-bit optical stream was measured, which can be mimicked by the corresponding four-bit inputs in the range “0000” to “1111,” as shown in Fig. 4f. Each periodic input waveform (0.5 s pulse width, 0.5 s pulse interval) is considered as one bit, in which the “off” and “on” states of the light pulse are represented as “0” and “1” in the time frames. The configuration of the input/output feature space is the basis for readout training. Therefore, all the *I*–*t* characteristics of all the four-bit inputs of the pixel sequences have been measured and sampled for feature values. In addition, similar statistical results (five cycles for each input) further validate the reliability and repeatability of the QD-NW reservoir (Fig. S17). The photoresponse characteristics and input–output feature extraction of input signals which can illustrate device-to-device variation during the encoding operation are also characterized. Statistical data for the four representative four-bit inputs from stochastically selected 20 QD-NW devices are shown in Fig. S18, indicating stable encoding behavior and minimal performance deviation across the array. These results support the robustness and uniformity of our in-sensor reservoir computing system. On the basis of the conspicuous difference, each frame sequence can be featured by current sampling to realize feature extraction, as shown in Fig. 4g. The raw video was preprocessed into 15 × 12 pixels per frame, with four frames per clip, to adapt the four-bit light pulse input, which reveals that the reservoir array successfully retains the characteristics of the four-frame action stream. By simulating the readout network training shown in Fig. 4h, the recognition accuracy of the “run” action successfully achieves 95% accuracy only after 20 training epochs. The recognition accuracy of all 10 human actions increased after training (Fig. 4i). As a result, we successfully constructed a physical reservoir computing paradigm in which a hardware system with tunable volatile memory and nonlinear readout dynamics serves as the reservoir.

### Robustness and Increased Recognition Accuracy of the QD-NW Sensor Enabled via Synergistic Amalgamation of the Two Photoresponse Modes

**Fig. 5:**
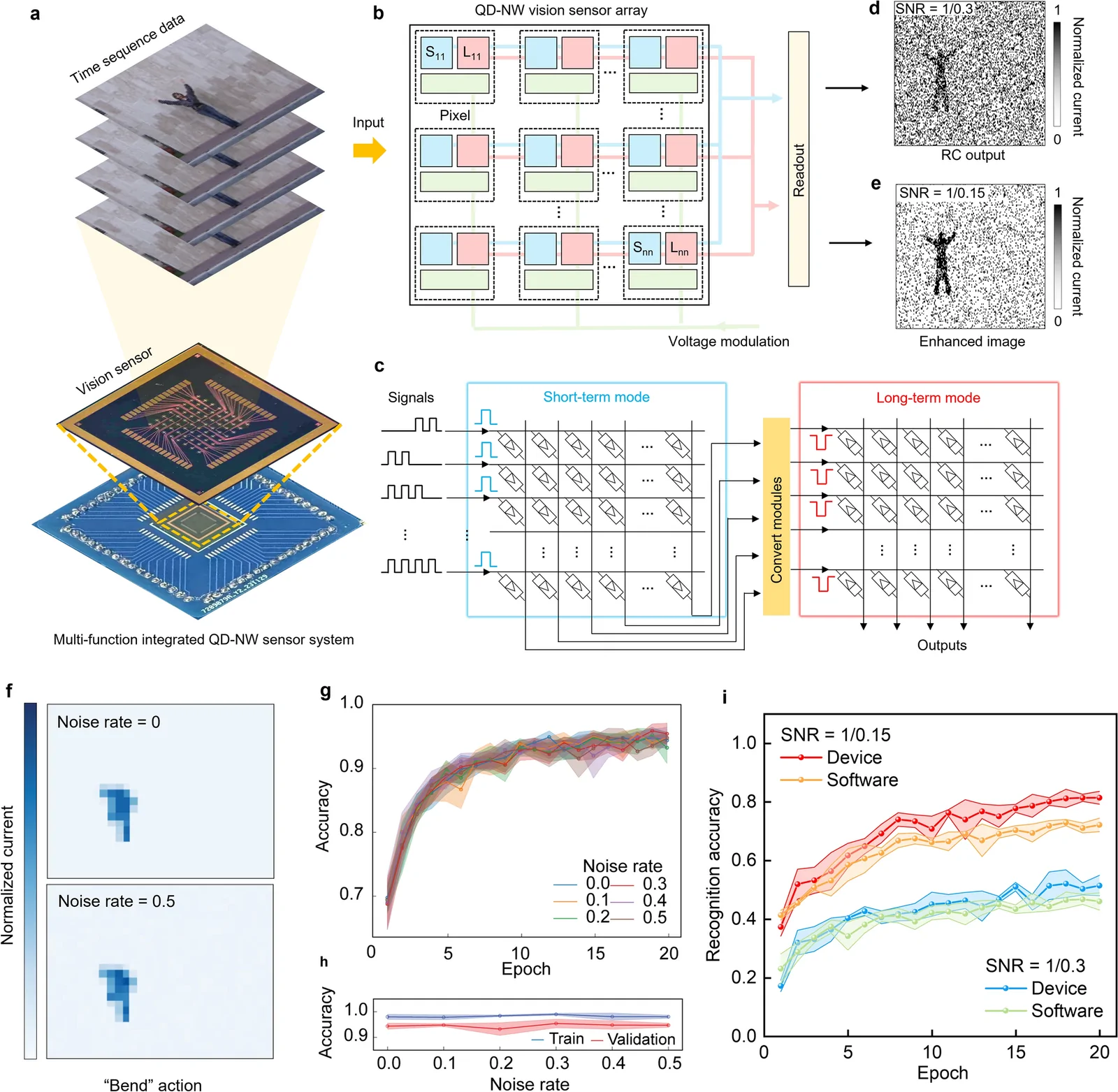
The integration of the two modes with great robustness results in higher recognition accuracy after in-sensor denoising. **a** Illustration of the time sequence image frame sensed by the QD-NW sensor array. **b** Schematic of the QD-NW sensor array, which consists of two modes in each pixel. **c** Circuit diagram of the sensor system. The input data are processed by the short-term mode-based RC system, which outputs the current and converts it into the inputs of the long-term mode section for image enhancement. **d** Extracted wave-two-hands action under input video SNR = 1/0.3. **e** Extracted wave2 action under input video SNR = 1/0.15. **f** Bend action in the HAR dataset readout current maps from NW-RC under Gaussian noise rates of 0.0 and 0.5 for simulating variations in NW reservoir output. **g** Validation accuracy versus training epochs of device output with noise rates ranging from 0.0 to 0.5. (Shaded area: std. *N* = 10). **h** Recognition accuracy after 20 training epochs of 10 human actions versus the device output noise rate. (Shaded area: std. *N* = 10). **i** Recognition accuracy comparison between SNRs of 0.3 and 0.15, corresponding to the scenarios of unenhanced and preprocessed images, respectively. (Shaded area: std. *N* = 5)

To illustrate the potential of our QD-NW sensor for high-accuracy human action classification, the conceptual configuration of the QD-NW vision sensor chip and the processing pathway are demonstrated. Figure 5a displays time sequence image frames captured by the sensor array, depicting the wave-two-hands action. The dynamic image sensor module comprises a short-term mode for reservoir computing and a long-term mode for image enhancement, which can be modulated by voltage bias (Fig. 5b). To explain the operation process more clearly, we constructed a circuit diagram of the QD-NW chip (Fig. 5c). The input data are first processed by the short-term mode-based RC system and converted into persistent photocurrent characters. The readout photocurrents are then processed and fed into a conceptual converter module that transforms the electrical output back into controlled optical pulses which serve as inputs for the second stage where the long-term PPC effect enables image enhancement. The flowcharts of the experimental procedure are shown in Fig. S19, and a detailed discussion is as follows.

A scenario with unavoidable input noise often exists in real-world video recognition tasks. On the basis of the aforementioned in-sensor processing feature and high-performance NW-RC system, the recognition accuracy of the human actions before and after contrast enhancement was evaluated. Figure 5d, e shows the extracted wave2 action under SNRs of 1/0.3 and 1/0.15, corresponding to the unenhanced and preprocessed video frames, respectively, indicating that the in-sensor preprocessing character evidently reduced the noise rate, improving the contrast between the target pixels and backgrounds.

Importantly, the robustness of the RC system plays a key role in the performance of the proposed artificial vision system. To estimate the robustness of the NW-RC system, different levels of device noise, characterized by cov (coefficient of variance) of Gaussian noise, were applied to the NW reservoir outputs to mimic the application of nonideal factors (e.g., device-to-device and cycle-to-cycle variations, operational discretization, etc.) on the hardware. Figure 5f depicts the bend action readout current maps from the NW-RC at noise rates of 0 (noise-free) and 0.5 (amplification level) (a flowchart is shown in Fig. S20). Notably, the recognition accuracy remains above 90% even at noise rates of up to 50%, as shown in Fig. 5g, and varying degrees of noise are introduced into the system to analyze the impact on accuracy. A comparison of the validation accuracy versus training epoch for noise rates of 0.0–0.5 (cov) is shown in Fig. S21. The recognition accuracy after 20 training epochs of 10 human actions is very close to the ideal value (Fig. 5h). These results suggest that the physical NW-RC system successfully extracts motion features from optical frame sequences with favorable robustness. In summary, the as-built in-sensor RC system leveraging our proposed nanowire synaptic devices has promising potential to be competent for high-precision video classification tasks.

The results of the noisy HAR recognition task are shown in Fig. 5i. We achieved a remarkable improvement in accuracy from 51.4% to 81.4% after integrating the in-sensor image enhancement function. Additionally, the recognition accuracy of the hardware-based RC system is higher than that of the software-only classification, further demonstrating the denoising capability of our device. Therefore, the integration of GaN nanowire neuro-inspired image enhancement and a robust reservoir computing system enables highly efficient and precise human action classification.

## Conclusions

In summary, we developed a versatile vision sensor composed of GaN/AlN-based ultrathin QD-NWs with configurable photoelectronic properties. The device exhibited remarkable voltage bias-assisted modulation capabilities, demonstrating two distinct modes of voltage bias-induced photoresponse: the “long-term mode” and the “short-term mode”. Under long-term mode, the photoresponse under negative bias enables effective image sensing and preprocessing, achieving high image enhancement corresponding to light dosage-dependent plasticity. Moreover, a high-performing and robust reservoir computing system is developed on the basis of the QD-NW sensor in short-term mode under positive bias, attaining impressive recognition accuracy for human actions. Notably, a significant increase in recognition accuracy is observed, increasing from 51.4% to 81.4% after preprocessing in long-term mode, indicating the highly synergistic integration of the two modes and the establishment of an efficient artificial vision system. The QD-NW-based bioinspired sensor enables neuromorphic hardware to achieve the perception and preprocessing of visual information in simple devices, facilitating the development of compact and efficient artificial vision systems in the future.

## Supplementary Information

Below is the link to the electronic supplementary material.

Supplementary file1 (DOCX 2412 kb)
