# Supplementary material for: Ultrathin Gallium Nitride Quantum-Disk-in-Nanowire-Enabled Reconfigurable Bioinspired Sensor for High-Accuracy Human Action Recognition
Source: Nanomicro Lett. 2025 Sep 1;18:54. doi: 10.1007/s40820-025-01888-w (PMC12401858; doi:10.1007/s40820-025-01888-w)
Supplement: Supplementary file 1 — Supplementary file1 (DOCX 2412 kb) [file 40820_2025_1888_MOESM1_ESM.docx]

Supporting Information for

**Ultrathin Gallium Nitride Quantum-Disk-In-Nanowire-Enabled Reconfigurable Bioinspired Sensor for High-Accuracy Human Action Recognition**

Zhixiang Gao^1,#^, Xin Ju^2,#^, Huabin Yu^1,#^, Wei Chen^1^, Xin Liu^1^, Yuanmin Luo^1^, Yang Kang^1^, Dongyang Luo^1^, JiKai Yao^1^, Wengang Gu^1^, Muhammad Hunain Memon^1^, Yong Yan^1^*, Haiding Sun^1^*

^1^ iGaN Laboratory, School of Microelectronics, University of Science and Technology of China, Hefei 230029, P. R. China

^2^ Institute of Materials Research and Engineering, 2 Fusionopolis Way, #08-03Agency for Science Technology and Research Singapore 138634, Singapore

^#^ Zhixiang Gao, Xin Ju, and Huabin Yu contributed equally to this work.

***Corresponding authors. E-mail: [yanyong365@ustc.edu.cn](mailto:yanyong365@ustc.edu.cn) (Yong Yan), [haiding@ustc.edu.cn](mailto:haiding@ustc.edu.cn) (Haiding Sun)

**Supplementary Figures**


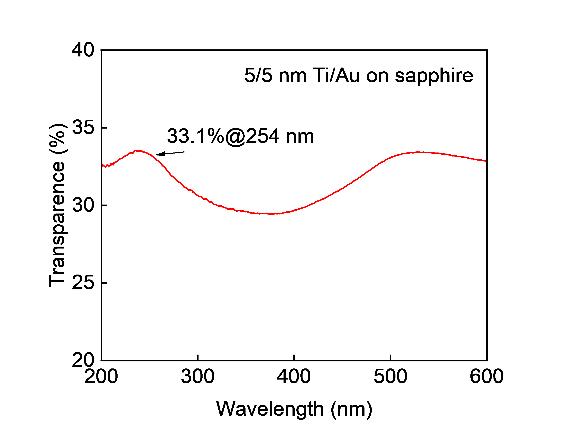


**Fig. S1** Wavelength-dependent transmittance curve of 5/5 nm Ti/Au on a sapphire substrate


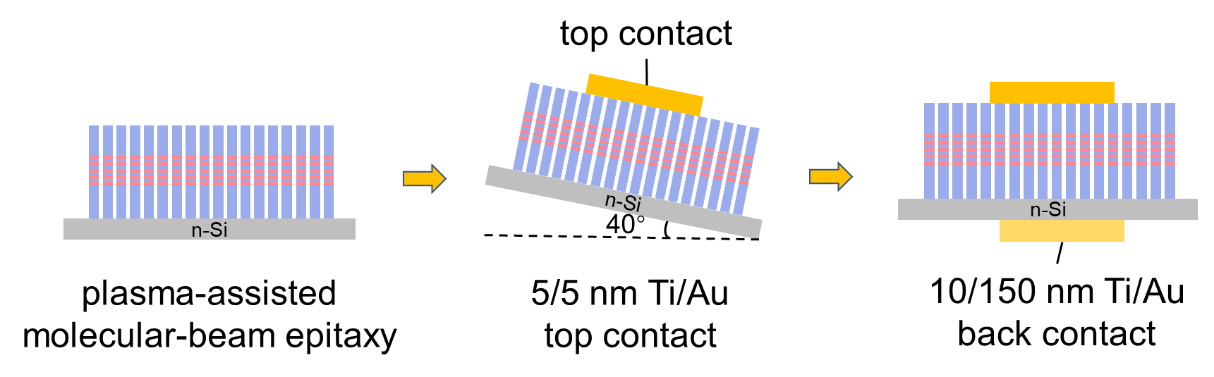


**Fig. S2** Fabrication process of the proposed nanowire optoelectronic device


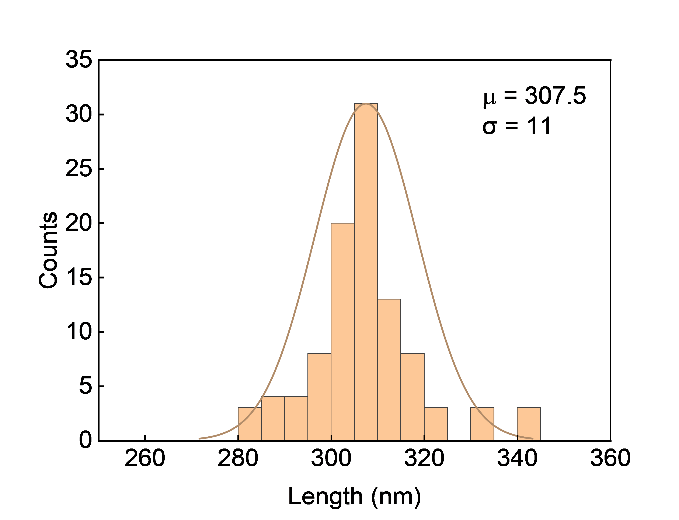


**Fig. S3** Statistics of length from 100 nanowires. The average length of the grown nanowire is ~300 nm with *μ* = 307.5 nm, *σ* = 11 nm, showing good uniformity


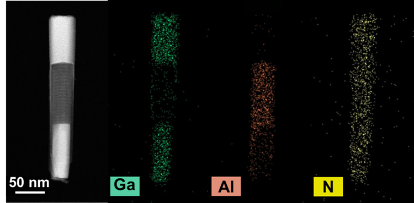


**Fig. S4** Energy-dispersive spectroscopy (EDS) element mapping of the nanowire


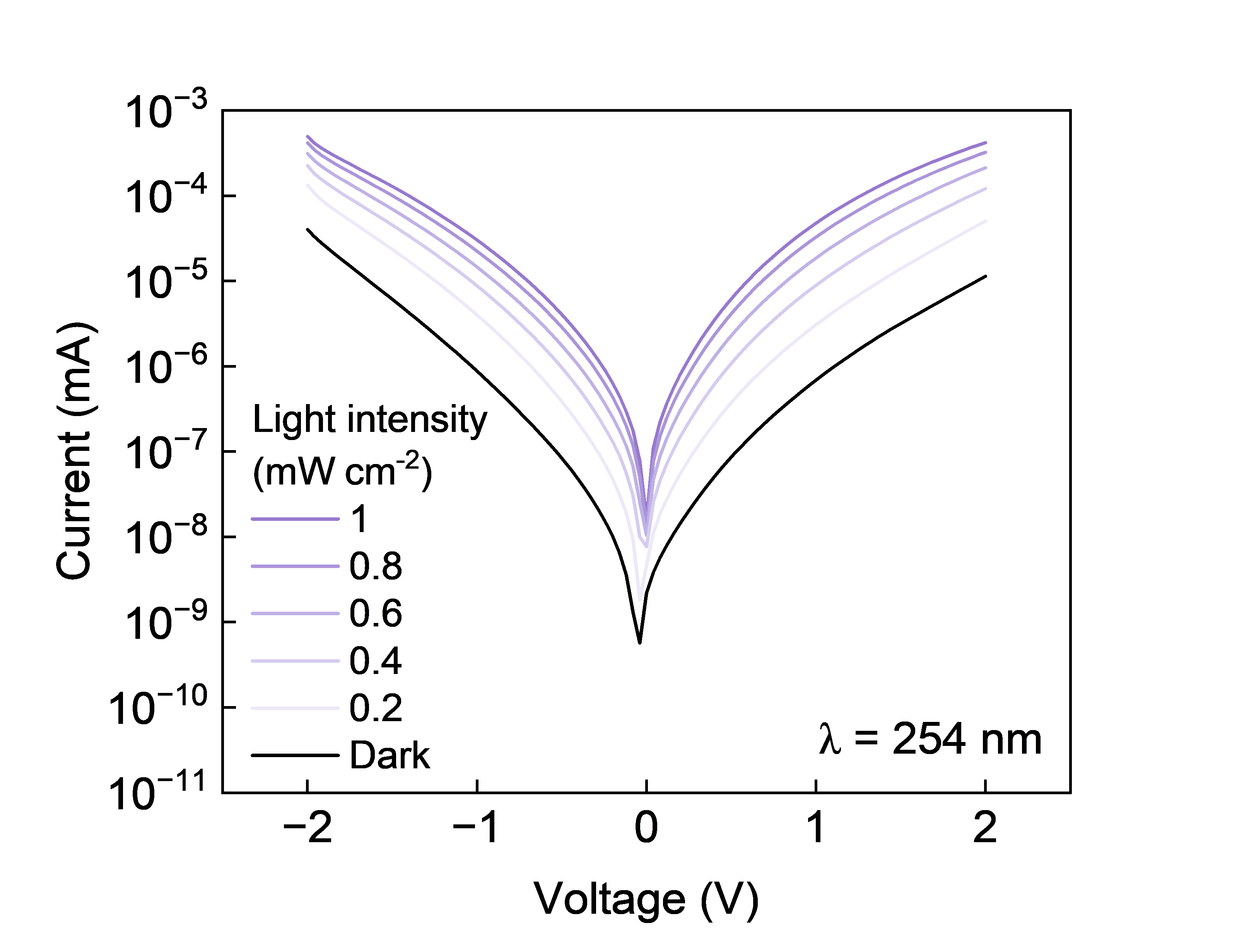


**Fig. S5** Current-voltage characteristics of a representative device under different light intensities


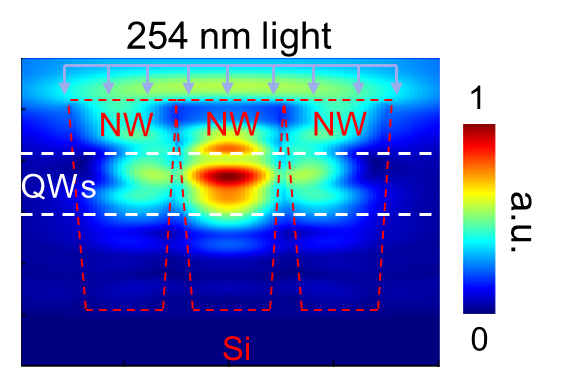


**Fig. S6** Cross-sectional optical field distribution of NWs array on Si substrate, illuminated by 254 nm light
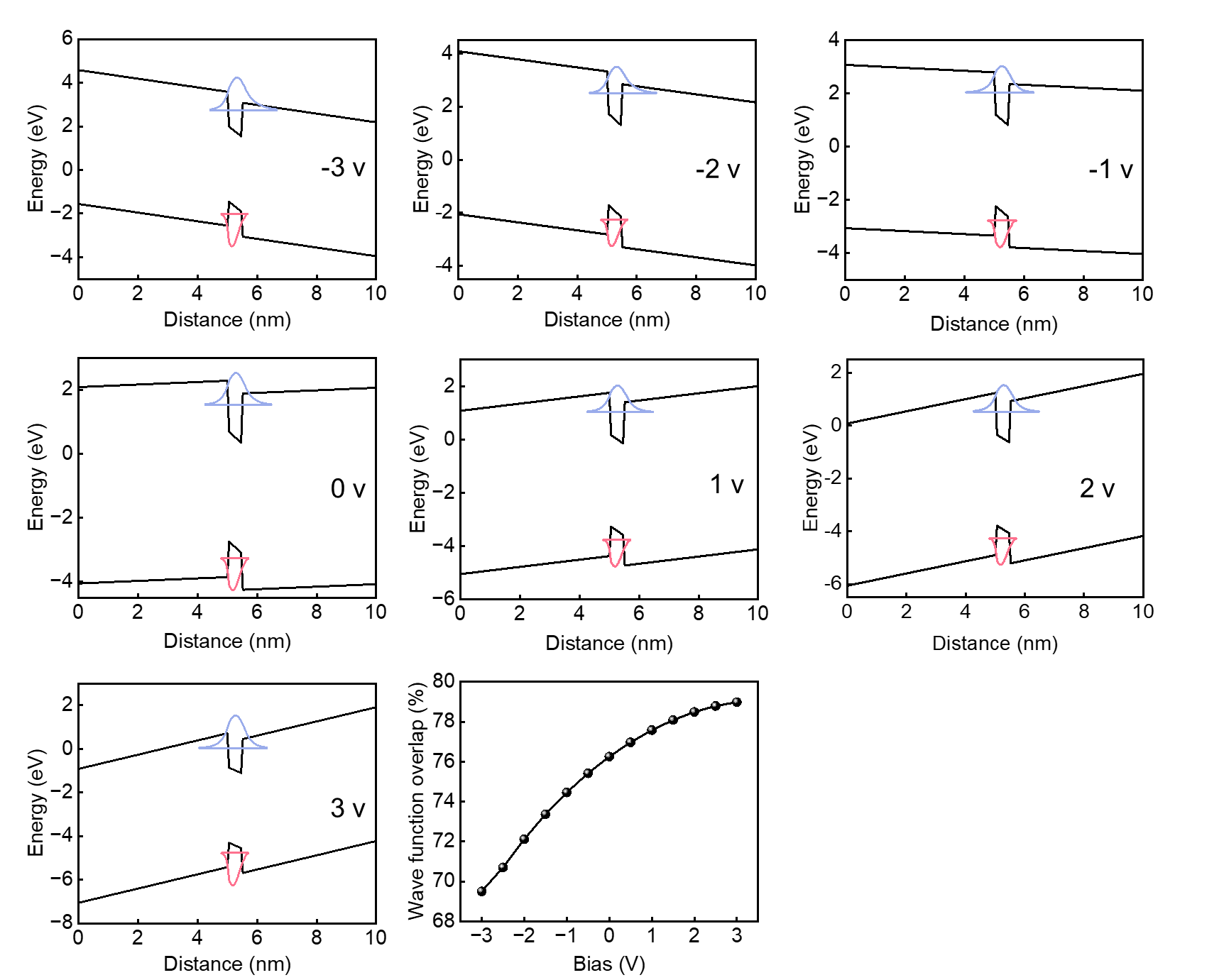


**Fig. S7** Band structure and wavefunction overlap under different bias conditions
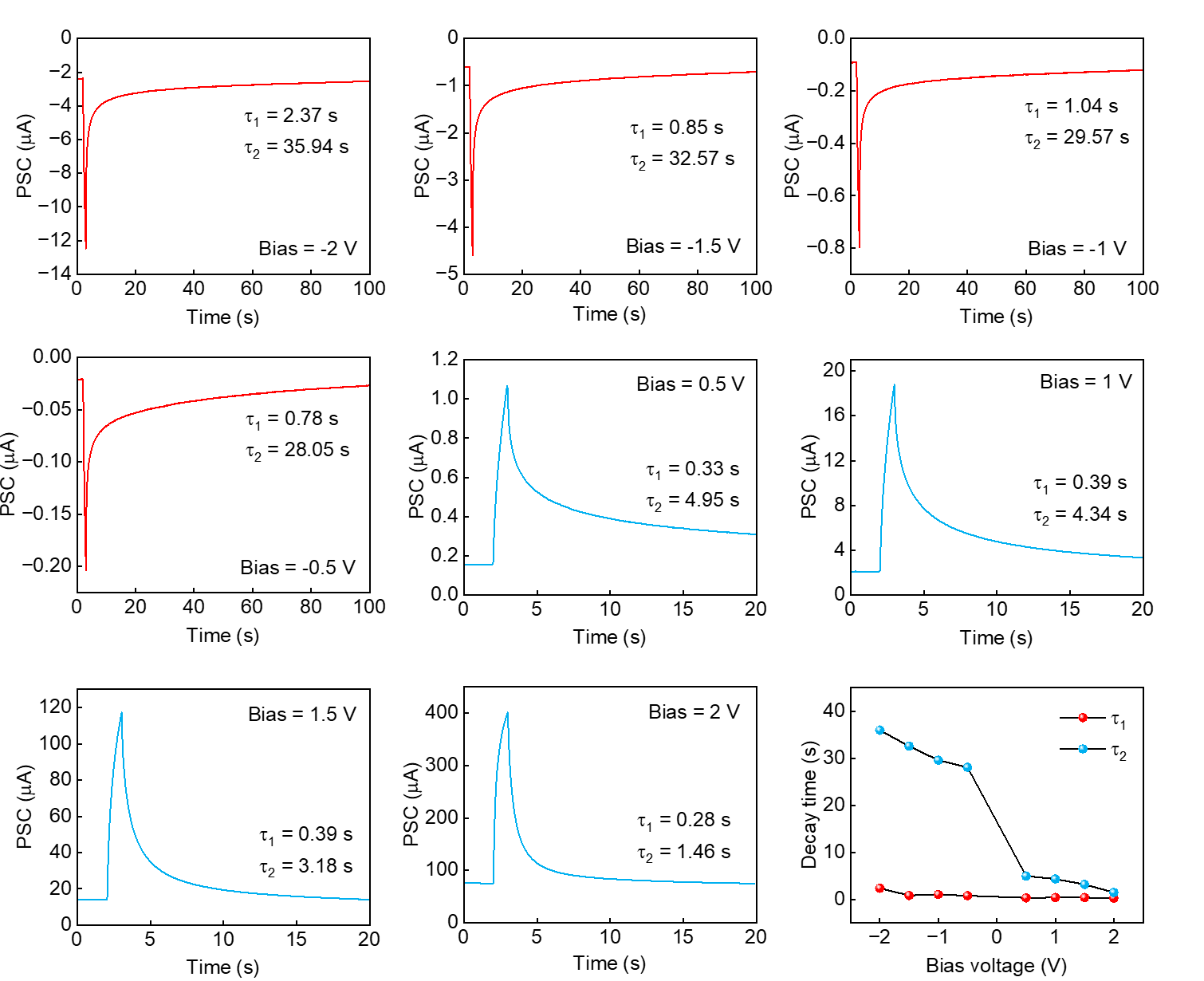


**Fig. S8** PPC characteristics extracted under reverse and forward bias with the same illumination conditions, showing an obvious difference in the fitting parameter
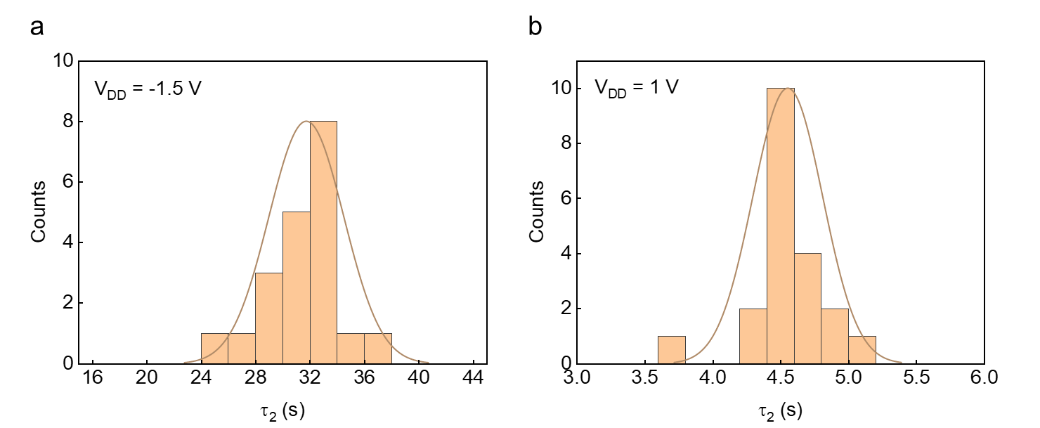


**Fig. S9** Statistic data of the PPC fitting parameter τ_2_ under (**a**) negative and (**b**) positive voltage bias from stochastically selected 20 QD-NW devices


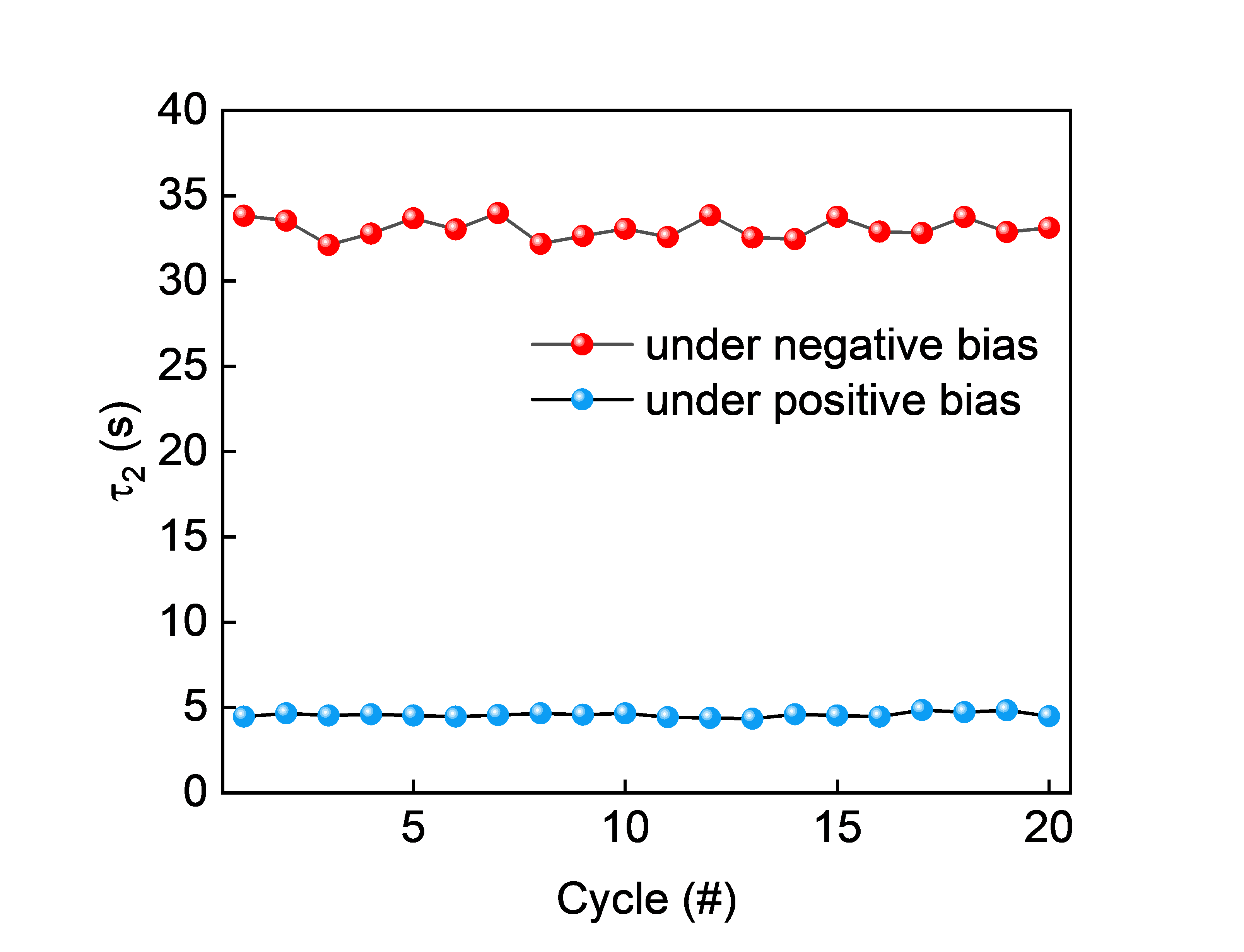


**Fig. S10** Statistic data of the PPC fitting parameter *τ*_2_ under negative and positive voltage bias sampled by 20 cycles from a representative device

**
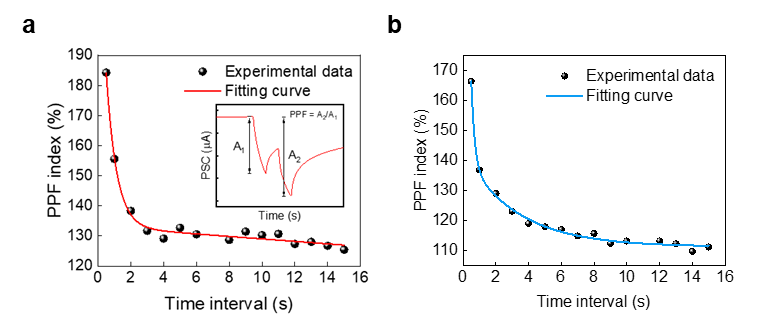
**

**Fig. S11 Modulation ratio of PPF index as a function of interval time (Δt).** (a) PPF index extracted under negative bias. The inset plots the time-resolved post-synaptic current of device at two successive light pulses. (b) PPF index extracted under positive bias


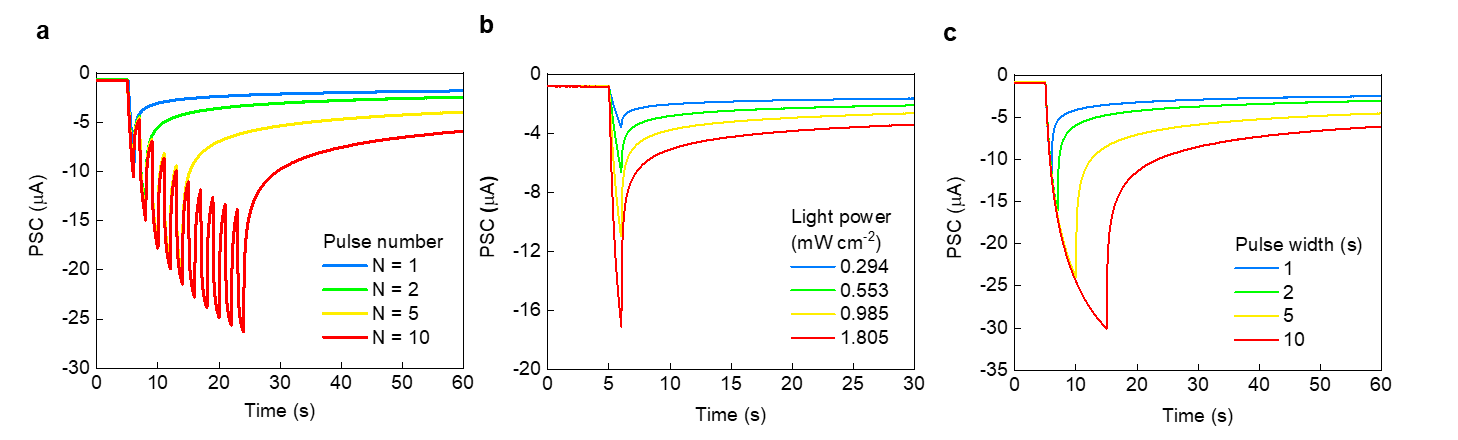


**Fig. S12 Synaptic plasticity transition under long-term mode.** (**a**) Pulse number dependent transition between short-term plasticity and long-term plasticity. (**b**) Emulation of STP and LTP behavior by light intensity-varied photonic stimuli. (**c**) Synaptic plasticity transition triggered by presynaptic pulses with different pulse durations


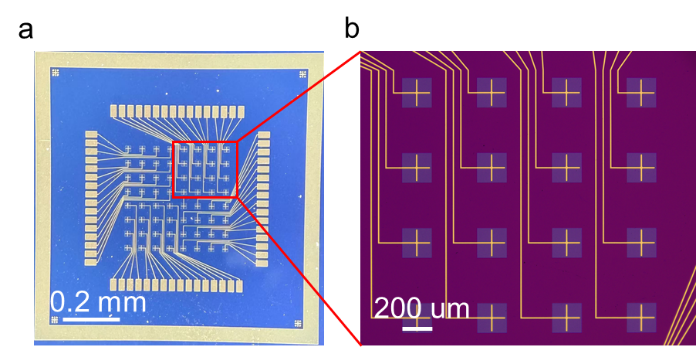


**Fig. S13** (**a**) Optical image of the 8×8 nanowire array. (**b**) Partial enlarged detail of the array


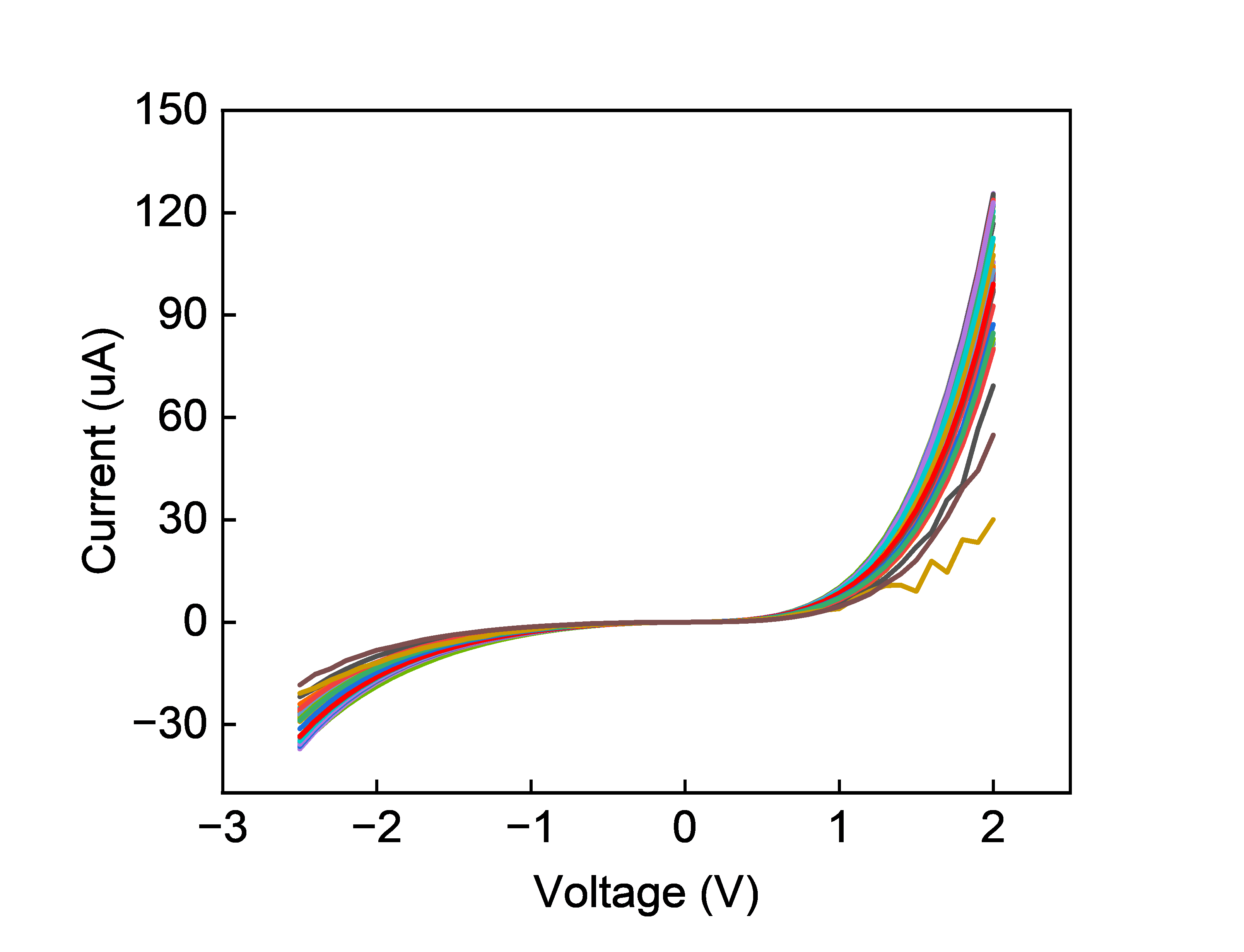


**Fig. S14** Device-to-device variation of the sensor array


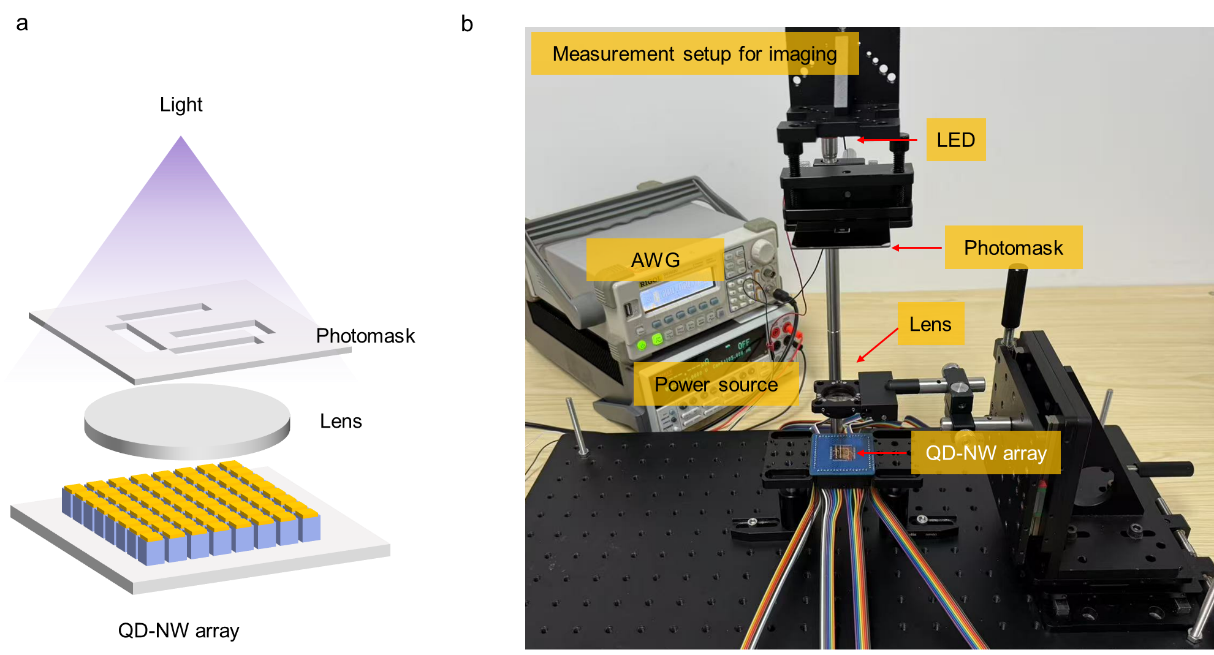


**Fig. S15** “G” shaped photomask with 17 pixels for imaging sensing


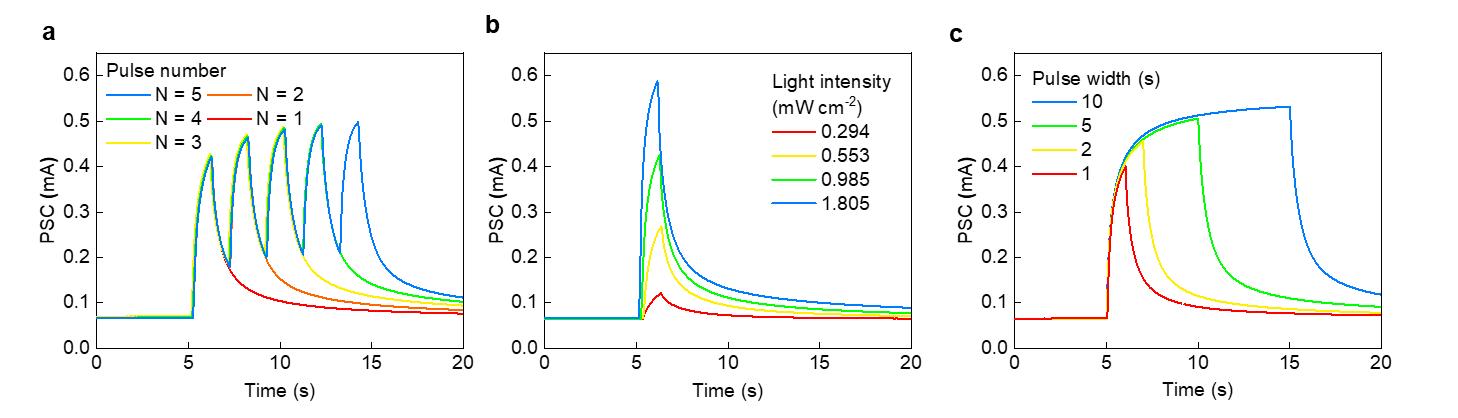


**Fig. S16** **Synaptic plasticity transition under short-term mode.** Transition between STP and LTP by adjusting the (a) pulse number; (b) light intensity; (c) pulse duration


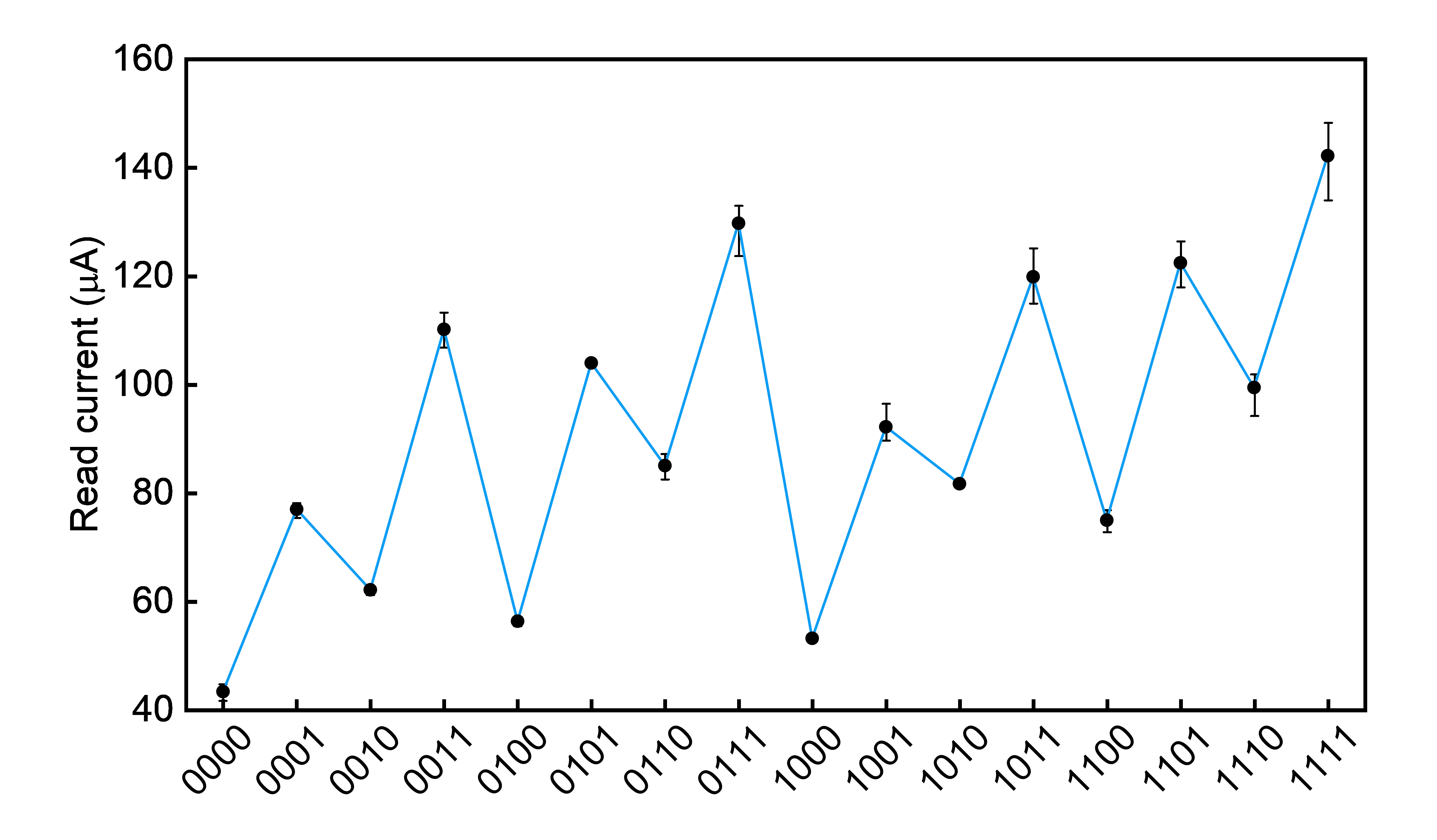


**Fig. S17** Reliability and repeatability of the QD-NWs device for reservoir computing, each input contains 5 cycles

**Fig. S18** Statistical data of four representative 4-bits inputs from 20 stochastically selected QD-NW devices under 1 mW cm^-2^ light condition


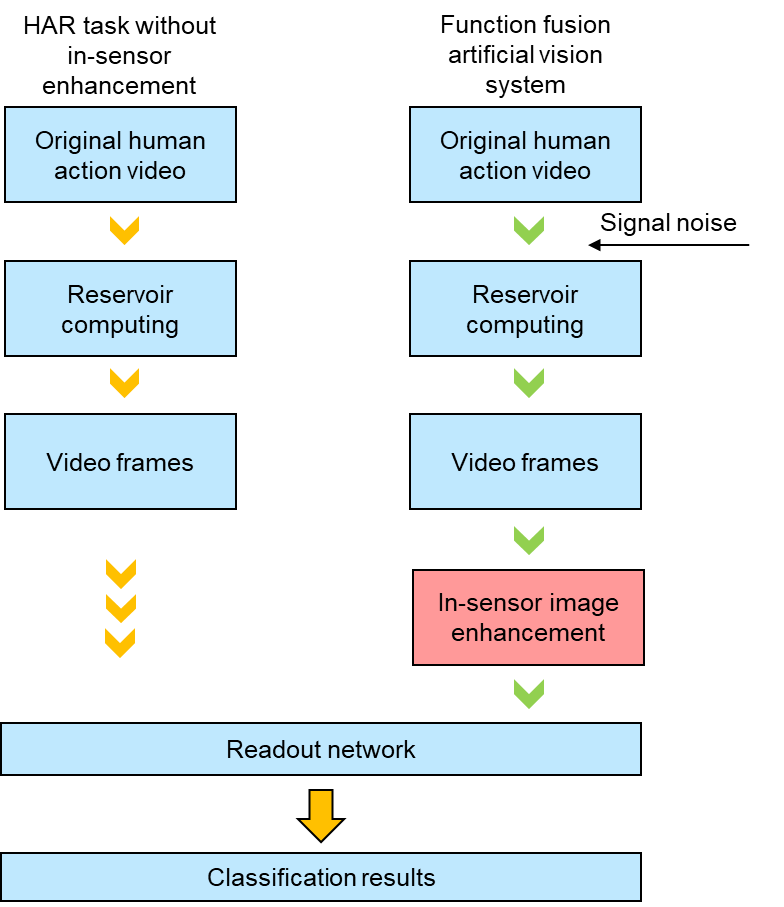


**Fig. S19** **Flowchart of the two functions fusion.** Flow 1 demonstrates the classification results with raw inputs. While flow 2 presents a preferable result with a pre-processing unit followed by RC


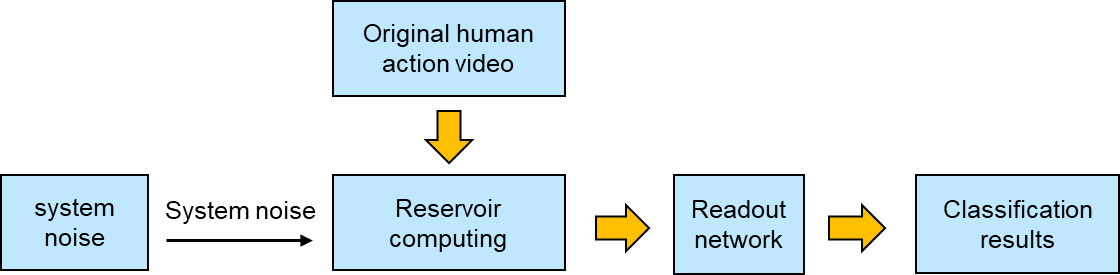


**Fig. S20** **Flowchart of the robustness verification.** To verify the robustness of the RC system, different levels of device noise are applied to the NW reservoir outputs to mimic the application of non-ideal factors on the hardware


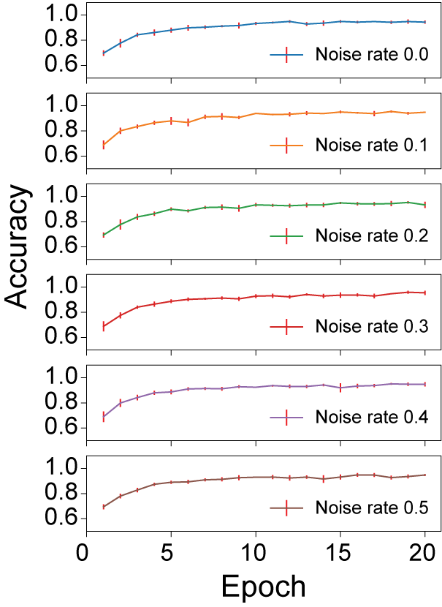


**Fig. S21** Comparison of validation accuracy versus training epoch for noise rates of 0.0 to 0.5 (*cov*), respectively
